# Supplementary figures and images for: Intima–Media Thickening with Carotid Webs: A Case Report of a Potentially High-Risk Association
Source: Diagnostics (Basel). 2025 Oct 30;15(21):2756. doi: 10.3390/diagnostics15212756 (PMC12610761; doi:10.3390/diagnostics15212756)

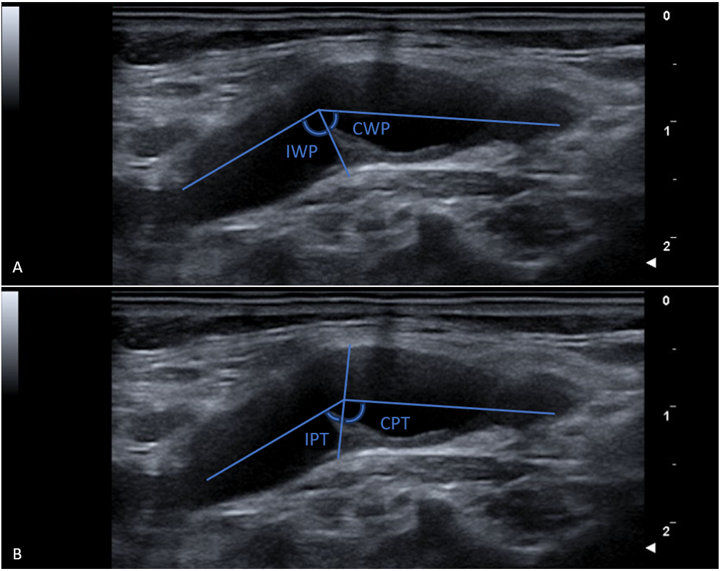

Supplement: Supplementary file 1 [file diagnostics-15-02756-s001.zip › diagnostics-3932716-supplementary.png]
